# Dermoscopy of Solitary Multinucleate Cell Angiohistiocytoma: A Rare Amelanotic Clinical Imitator of Melanoma

**DOI:** 10.7759/cureus.84796

**Published:** 2025-05-25

**Authors:** Georgios Sarris, Zannis Almpanis, Zoe Apalla, Alexander Katoulis, Dimitrios Sgouros

**Affiliations:** 1 2nd Department of Dermatology and Venereology, “Attikon” General University Hospital, National and Kapodistrian University of Athens, Athens, GRC; 2 Department of Pathology, “Pathlabs” Pathology Laboratory, Athens, GRC; 3 2nd Department of Dermatology, Aristotle University of Thessaloniki, Thessaloniki, GRC

**Keywords:** amelanotic melanoma, dermatofibroma, dermoscopy, multinucleate cell angiohistiocytoma, skin cancer

## Abstract

Multinucleate cell angiohistiocytoma (MCAH) is a rarely reported and likely underdiagnosed cutaneous entity with benign biological behavior. MCAH is described as a solitary or multiple proliferation of vascular and fibrohistiocytic origin, with largely unknown etiopathogenesis. It presents as red-brown to violaceous papules or nodules, mainly on the extremities, and is usually asymptomatic. A definitive diagnosis of MCAH requires specific histopathological and immunohistochemical findings. The following report presents a case of solitary MCAH with the aim of clarifying the clinical, histological, and especially dermoscopic aspects of this dermatological disorder, which may resemble more aggressive skin tumors.

## Introduction

Multinucleate cell angiohistiocytoma (MCAH) is a benign cutaneous proliferation of vascular and fibrohistiocytic origin, first described as a distinct entity by Smith N and Wilson JE in 1985 [1,2]. Published cases remain extremely limited, approximately 140 by 2020, as the rarity of MCAH, its frequent misclassification as other types of dermatofibroma, and the subsequent lack of clinical suspicion contribute to underdiagnosis. Clinical manifestations typically include asymptomatic, well-circumscribed papules or plaques of reddish-brown to violaceous color, occurring either as solitary or multiple lesions [2-4]. Pathological features such as small vascular proliferations with round endothelial cells, fibroblasts with radial morphology, and angulated multinucleate cells (MCs) are considered pathognomonic [3]. The etiopathogenesis of MCAH remains largely unknown, although a possible correlation with immunological abnormalities and reactive responses has been suggested [2,3]. In the following case report, we describe a lesion with dermoscopic characteristics that mimicked amelanotic melanoma. Thorough analysis and correlation of available case data with current literature were essential to establishing the diagnosis of this rare, benign, and recently recognized disorder.

## Case presentation

**Figure 1:**
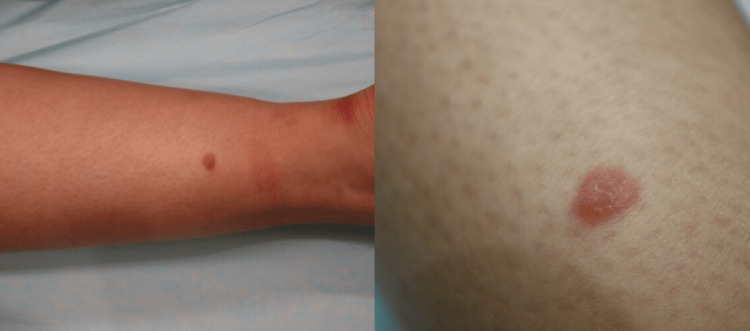
Clinical image showing a pink nodule measuring 8 mm in diameter on the posterior aspect of the left shin.

**Figure 2:**
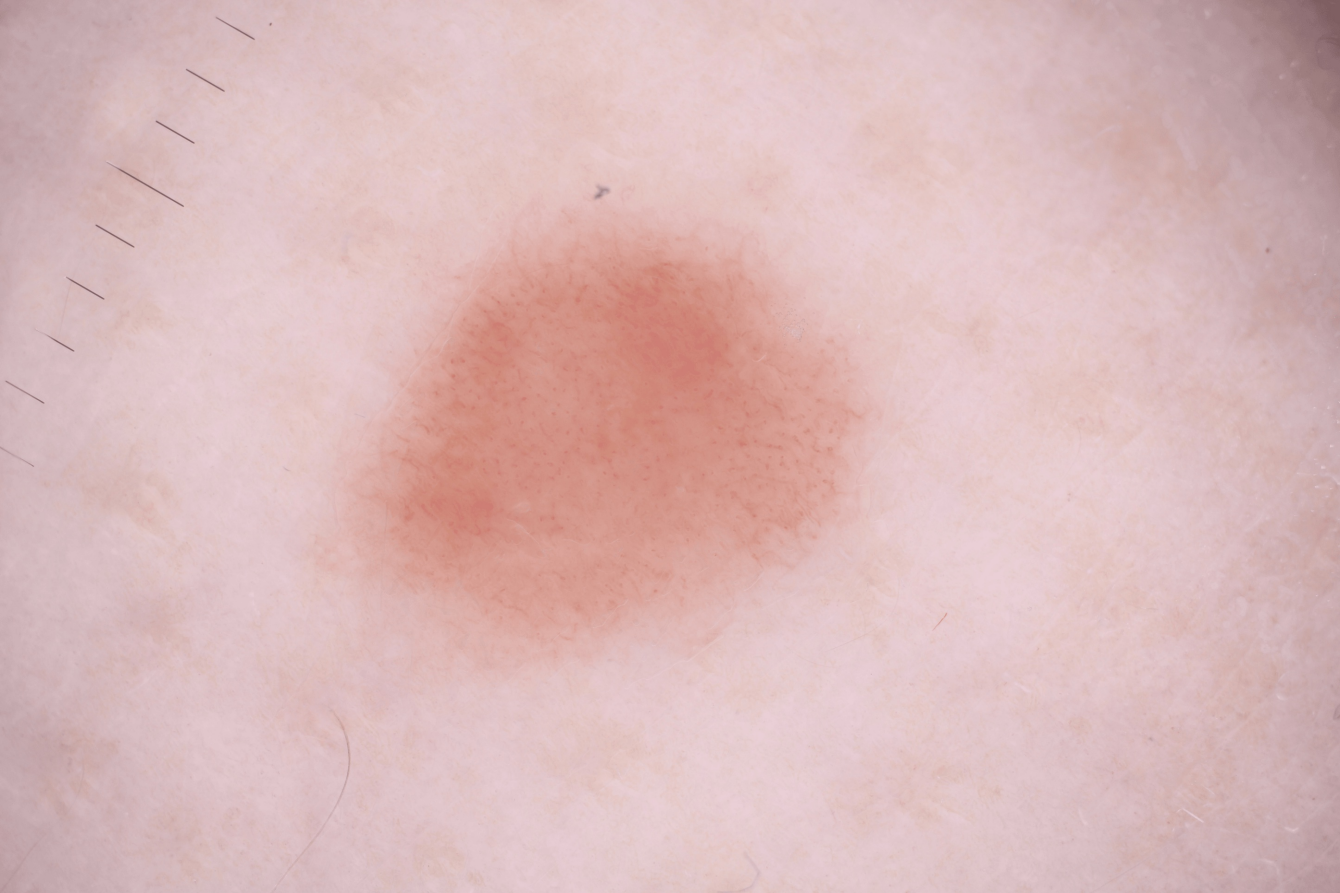
Dermoscopic image of the lesion showing randomly arranged dotted vessels and short linear telangiectasias on a faint brown background.

A 32-year-old Caucasian female presented with an eight-month history of a clearly defined, pink, asymptomatic nodule measuring 8 mm in diameter. The lesion was of medium firmness and located on the posterior surface of the left distal shin (Figure 1). The patient’s medical history was unremarkable. Dermoscopically, a dotted vessel pattern along with thin, linear, dilated vessels was observed, raising suspicion of nodular melanoma (NM) (Figure 2). Additionally, pale reddish and hypopigmented multifocally distributed areas were detected. Clinical and dermoscopic differential diagnoses included pseudolymphoma, mastocytoma, cutaneous lymphoma, Spitz nevus, lupus erythematosus tumidus, and NM.

**Figure 3:**
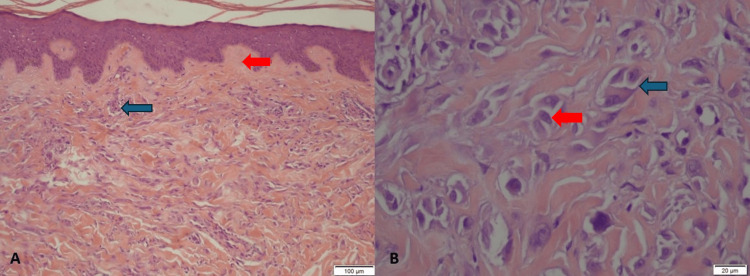
Histopathological features of the lesion. A: Prominent perivascular and interstitial dermal infiltrate with fibroblastic hyperplasia and early-stage thickening of collagen fibers in the papillary dermis (blue arrow). Fibrosis parallel to the surface in the upper dermis (red arrow) is due to fibrohistiocytic proliferation, with thickened vessels displaying endothelial cells protruding into the vascular lumina. Mild perivascular inflammation with multinucleate histiocytes and sparse plasmocytes is also observed (H&E, intermediate magnification ×10). B: Multinucleated histiocytic cells with angular or scalloped cytoplasm and 3 to 10 hyperchromatic nuclei (blue arrow). Bizarre multinucleated fibroblasts with at least two nuclei (red arrow) (H&E, high magnification ×40).

A 4-mm punch biopsy was performed, which histologically demonstrated fibrohistiocytic proliferation in the dermis with vessels exhibiting endothelial cells protruding into the vascular lumina. A mild perivascular inflammatory response was also present, featuring multinucleate histiocytes and sparse plasmocytes in the infiltrate. In the papillary dermis, fibroblastic hyperplasia and thickening of collagen fibers at an early stage were observed. The histological evaluation favored the diagnosis of MCAH (Figure 3). Considering the benign nature of MCAH, the patient opted not to undergo treatment.

## Discussion

There is sufficient clinical and histopathological evidence to support that MCAH represents a distinct entity, although it has been suggested that MCAH should be classified as a type of dermatofibroma [4]. MCAH is rarely reported, primarily due to a lack of familiarity among clinicians and pathologists with this type of proliferation [3].

Spontaneous regression of MCAH is rare, and in the majority of cases, it follows a persistent, slowly progressive course [4]. A predilection for middle-aged to elderly women had been reported in the past; however, recent findings suggest no significant gender association [3,5]. Most cases appear between the 5th and 8th decades of life, with a significantly lower mean patient age for solitary compared to multiple MCAH presentations [3].

Common sites of involvement include the dorsum of the hands and fingers, arms, shoulders, legs, or feet, while a small minority occur on the trunk or face [3]. Typically, MCAH presents as clearly defined papules or, less frequently, nodules ranging between 2 and 15 mm in diameter, following a random, linear, or annular distribution [2]. A few cases of disseminated and giant plaque-type MCAH have also been described [2,6].

The etiopathogenesis of MCAH remains largely unknown, but it is generally accepted that MCAH results from a reactive, rather than neoplastic, process. Consistent reports of benign lesions and a silent clinical course, with no malignant transformation or extracutaneous involvement, support this view [7]. MCAH is thought to involve the proliferation of venules, capillaries, and dendritic cells in the dermis, along with a lymphocytic infiltrate and multinucleated cells (MCs) [4].

Four diagnostic criteria have been proposed for MCAH: (a) bizarre multinucleated fibroblasts with at least two nuclei; (b) fibrosis parallel to the surface in the upper dermis; (c) presence and thickening of vessels superficially in the papillary dermis; and (d) absence of perifollicular fibrosis [3].

The MCs that characterize MCAH exhibit angular or scalloped cytoplasm, abundant rough endoplasmic reticulum, and 3 to 10 hyperchromatic nuclei with reinforced nuclear membranes (“zonula nucleus occludens”) located peripherally within the cells. MCs are believed to have a fibrohistiocytic origin, as their immunohistochemical profile lacks monocyte/macrophage markers [4].

Regarding the dermoscopic features of MCAH, the main findings reported in the literature include asymmetrical, vaguely demarcated reddish areas, presumably linked to dilated vessels, whitish areas associated with collagen thickening, and fine isolated reticulated areas considered to correspond to melanin in the epidermal ridges. Additional features include dotted vessels reflecting discrete small-vessel hyperplasia and an irregular surface with small, arborizing fissures [8,9]. In our case, randomly arranged dotted vessels were observed, along with short linear telangiectasias on a faint brown background, a dermoscopic feature shared with dermatofibroma. Dotted vessels in a nodule are a diagnostic clue for Spitz nevus and amelanotic NM, which can be particularly insidious in its early stages and therefore easily overlooked [10]. Thus, solitary MCAH should be considered in the differential diagnosis of amelanotic NM. Moreover, our observations align with the literature regarding the presence of thin telangiectatic vessels and pale-reddish areas, which may be attributed to the superficial thickening of vessels in the papillary dermis. On the other hand, we also describe areas of light hypopigmentation, likely correlated with superficial dermal fibrosis arranged parallel to the skin surface. This finding serves as a distinguishing feature from dermatofibroma, where the orientation of fibrosis is typically random rather than parallel to the epidermis [3]. Additionally, as previously noted, the white coloration typically observed in the dermoscopy of several clinical entities (e.g., dermatofibroma, melanoma, basal cell carcinoma) is generally linked to a fibrotic reaction in deeper dermal structures and should be differentiated from hypopigmentation.

## Conclusions

To conclude, based on our observations clarifying the pathological and dermoscopic characteristics of MCAH, the solitary form of this disorder does not merely represent another variant of “vascular dermatofibroma,” as evidenced by the presence of histopathological horizontal dermal fibrosis and multinucleated fibroblasts. A detailed dermoscopic evaluation, alongside comparison with vascular proliferations and overlapping features with Spitz nevus and amelanotic NM, was essential for accurately assessing diagnostic probability and reaching a diagnosis with reasonable certainty. Raising clinician awareness of this potential clinical imitator of amelanotic melanoma, Spitz nevus, or dermatofibroma may broaden diagnostic insight and improve the overall management of non-pigmented solitary lesions of ambiguous nature, thereby reducing unnecessary excisions. Enhanced clinical suspicion of MCAH may help decrease underdiagnosis, increase the number of reported cases, and ultimately enable a more comprehensive understanding of this entity in the future.
